# A non-uniform allowance allocation method based on interim state stiffness of machining features for NC programming of structural parts

**DOI:** 10.1186/s42492-018-0005-2

**Published:** 2018-09-05

**Authors:** Sen Jiang, Yingguang Li, Changqing Liu

**Affiliations:** College of Mechanical and Electrical Engineering, Nanjing University of Aeronautics and Astronautics, Nanjing, China

**Keywords:** Machining features, NC programming, Interim state, Non-uniform allowance, Stiffness

## Abstract

For thin-walled parts, uniform allowance to each machining surface is allocated by the traditional machining method. Considering the sequence of the adjacent machining features, it may cause poor stiffness for some side walls due to a minor wall thickness, which may cause the deformation of the final formed parts to be large, or deduce machining efficiency for some machining features due to too thick remains. In order to address this issue, a non-uniform allowance allocation method based on interim state stiffness of machining features for the finishing of thin-walled structural parts is proposed in this paper. In this method, the interim state model of machining features is constructed according to the machining sequence of the parts, and the stiffness of the side wall is taken as the evaluation index to allocate reasonable allowance value to the corresponding machining surface to ensure the stiffness requirement of the parts in the machining process. According to the finite element simulation results, the non-uniform allowance allocation method proposed in this paper can effectively improve the stiffness of the parts and reduce the deformation of the parts, when compared with the traditional uniform allowance machining method.

## Background

**Fig. 1:**
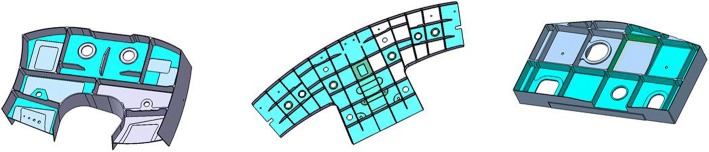
Typical thin-walled aircraft structural parts

Thin-wall structure is widely used in aircraft structural parts, as shown in Fig. 1. For this kind of parts, the structure is complex and the machining accuracy requirement is high. In order to ensure that the final deformation of thin-walled parts is within machining tolerance, it is necessary to meet the specific requirement of the part stiffness in the process of machining, especially in the finishing stage of the parts. According to the traditional uniform allowance programming method, all machining surfaces will be allocated the same allowance when finishing, and the effect of processing sequence among the processing features is not considered in this process. In fact, the wall thickness of the adjacent machining surface of the adjacent machining features is different when it is processed successively due to the machining sequence difference, and the stiffness of the post-processing feature may be insufficient when it is processed. Consequently, the deformation of the parts may be out of specification, or even the final parts may be scrapped.

### Literature review

When thin-walled parts being machined by NC machining, it is difficult to meet the design requirements because of poor stiffness and the deformation caused by various machining factors. Therefore, NC machining of thin-walled parts is a bottleneck problem in the machining field. Aiming at this problem, many scholars and technicians all over the world have tried and studied the corresponding methods, which can be roughly divided into the following four categories.

### Optimization of processing technology route

This kind of method is trying to eliminate the machining stress caused by cutting, clamping and the residual stress of the material itself by setting one or more semi-finishing and aging processes between rough machining and finishing. It is expected that the deformation of the material only occurs in the final finishing stage. Ren et al. [1] presented an efficient rough machining process for engine blade. In order to prevent the large cutting stress and deformation in rough machining, they carried out the method of dispersing the working processes, executing aging treatment and benchmark repairing several times to reduce the deformation caused by stress.

**Optimization of clamping process:** This kind of method attempts to ensure the stiffness of parts in machining process by changing the clamping forms, fixture tools and clamping force, so as to ensure the quality of parts. Wang et al. [2] presented a special fixture for thin-wall parts machining clamping by using low shrinkage and low melting point alloy. The experimental results show that high material removal rate and machining accuracy can be obtained by using this special fixture during the machining process. Considering the influence of clamping sequence on machining accuracy, Qin et al. [3] established a model for analyzing and optimizing clamping sequence of parts. This method can reduce the machining deformation and positioning error by establishing the relation between the distribution of contact force and the machining accuracy of the parts, and finally improve the machining rigidity of the system. Pan et al. [4] constructed the relationship model between clamping point contact force and contact deformation by finite element method, meanwhile, the nonlinear relationship between contact force, deformation and contact region is quantitatively analyzed, which provides a basis for the analysis of machining deformation and assembly error of thin-walled parts. Vol [5] proposed an optimization method of the clamping position and clamping point number based on genetic algorithm. The experimental results show that the method can improve the size and shape accuracy of machining parts. Xue et al. [6] put forward a synchronous optimization method of clamping force and cutting parameters based on genetic algorithm and finite element analysis to reduce machining deformation of parts. Selvakumar et al. [7] proposed an experimental design method based on artificial neural network. This method can reduce the clamping force and maximize the elastic deformation in the machining process by optimizing the clamping layout.

### Optimization of cutting force and error compensation

This kind of method is trying to reduce the deformation and vibration in the machining process by optimizing the cutting force and error compensation to ensure the forming quality of the final part. Qu et al. [8] proposed a multi-objective optimization method to minimize cutting force and maximize machining efficiency. Yang et al. [9] established the mathematical model of cutting force for thin-walled parts of titanium alloy by means of multiple regression and orthogonal test. Moreover, the influence of cutting force on machining deformation has been analyzed, which provides a reference for optimization of cutting parameters of difficult-to-machine materials. Zhou et al. [10] studied the nonlinear dynamic behavior of cutting force during the machining process of thin plate parts, and the results indicate that the cutting force can develop complicated dynamic behavior due to the time-delay effect. Huang et al. [11] used spectrum analysis and wavelet analysis to analyze and compare the cutting force and spindle acceleration signal between thin-walled and non-thin-walled titanium alloy parts to determine the influence of tool wear on cutting parts. Liu et al. [12] proposed a finite-element-based milling error prediction method. This method establishes the dynamic model of the tool in the whole continuous cutting process to obtain the moving state of the tool at any point. Then, the machining error of the parts surface can be obtained. Chen et al. [13] presented a method of active compensation for machining error of thin-walled parts. The method establishes a multi-layer cutting deformation model of thin-walled parts and realizes the compensation of machining error by layer-by-layer iterative calculation. Cui et al. [14] proposed a software error compensation method by reconstructing NC program. The experimental results indicate that the reconstruction of NC program can effectively improve the motion accuracy of NC machine tools. Liu et al. [15] proposed a comprehensive compensation method for machining errors of aircraft thin-walled parts. The method acquires the contour information of parts by mechanical scanning method and then establishes a unified target for model reconstruction. The experimental results show that the dimension feedback technique can effectively improve the machining quality and efficiency. Zhang et al. [16] proposed an adaptive compensation method for blade machining accuracy based on machine measurement. Simultaneously, an adaptive geometric model of machining parts was established by analyzing and comparing the calibration model with the real measurement data. It is used to accurately describe the machining accuracy of thin-walled blade with compound error compensation.

### Introduction of non-traditional processes

With the development of machining technology, some non-traditional machining techniques have been applied to the numerical control machining of thin-walled parts, among which ultrasonic vibration cutting and high-speed cutting are the most representative. Jiao et al. [17] have studied ultrasonic vibration machining. The results show that elliptical vibration cutting has many advantages: when the vibration frequency and amplitude increase, the cutting force and cutting temperature will decrease, and the surface quality of the parts can be improved accordingly, at the same time, the burrs caused by machining are limited to specific regions, which is suitable for thin-walled parts. Shamoto et al. [18] has developed a kind of TDF (Three Degree of Freedom) ultrasonic vibration equipment, which can produce arbitrary ultrasonic elliptical vibration in three-dimensional space, and it is very suitable for NC machining of 3D complex thin-walled parts. Nath et al. [19] put forward the concept of contact rate in ultrasonic vibration machining by studying parameters vibration frequency, vibration amplitude and cutting speed. The experimental results show that contact rate has an important influence on cutting force and tool wear. Yin et al. [20] designed a kind of single-drive ultrasonic elliptical vibration cutting equipment. The experimental results show that the device can reduce the cutting force and the surface roughness of the parts. By modifying the Cook model and simulating the cutting force, cutting shape, effective stress and cutting temperature of thin-walled parts, Tang et al. [21] established a finite element simulation model for high-speed milling, which provides a new idea for the deformation control of thin-walled parts.

The above methods can improve the machining quality of thin-walled parts from the aspects of machining process, clamping, fixture tool, error compensation, etc. However, few studies have focused on the structural characteristics and machining stiffness optimization of thin-walled parts.

**Fig. 2:**
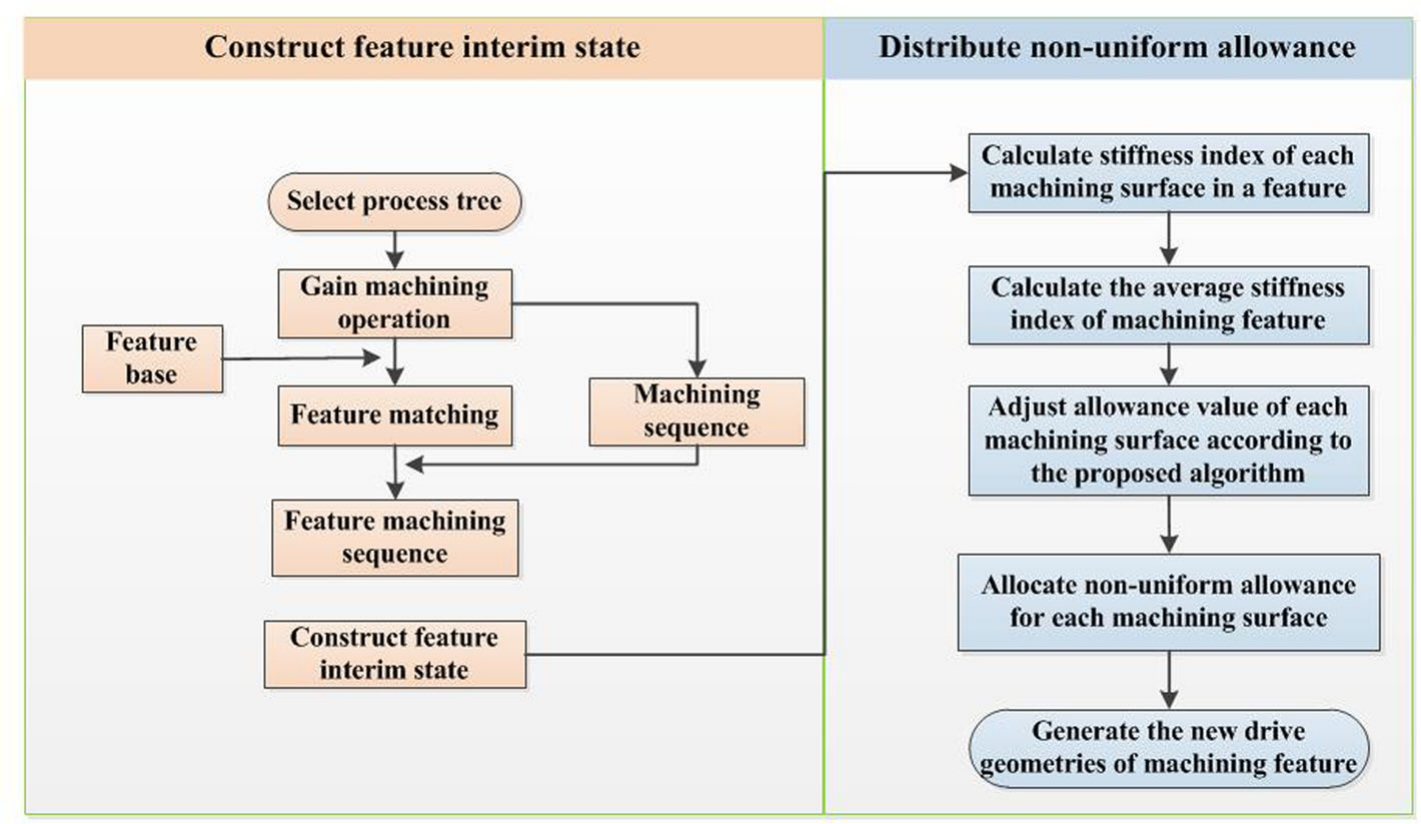
Basic idea of the proposed method

In order to solve the deformation problem caused by the lack of local stiffness of thin-walled parts caused by uniform allowance programming, a non-uniform allowance allocation method based on interim state stiffness of machining features is proposed in this paper. In this method, the interim state model of machining features is constructed according to the machining sequence of parts, and the stiffness of the corresponding machining surface is taken as the measuring index to assign reasonable finishing allowance value to the machining surface. The basic idea of this method is shown as Fig. 2.

## Methods

### Evaluation method of interim state stiffness of machining features

#### Interim state of machining features

**Fig. 3:**
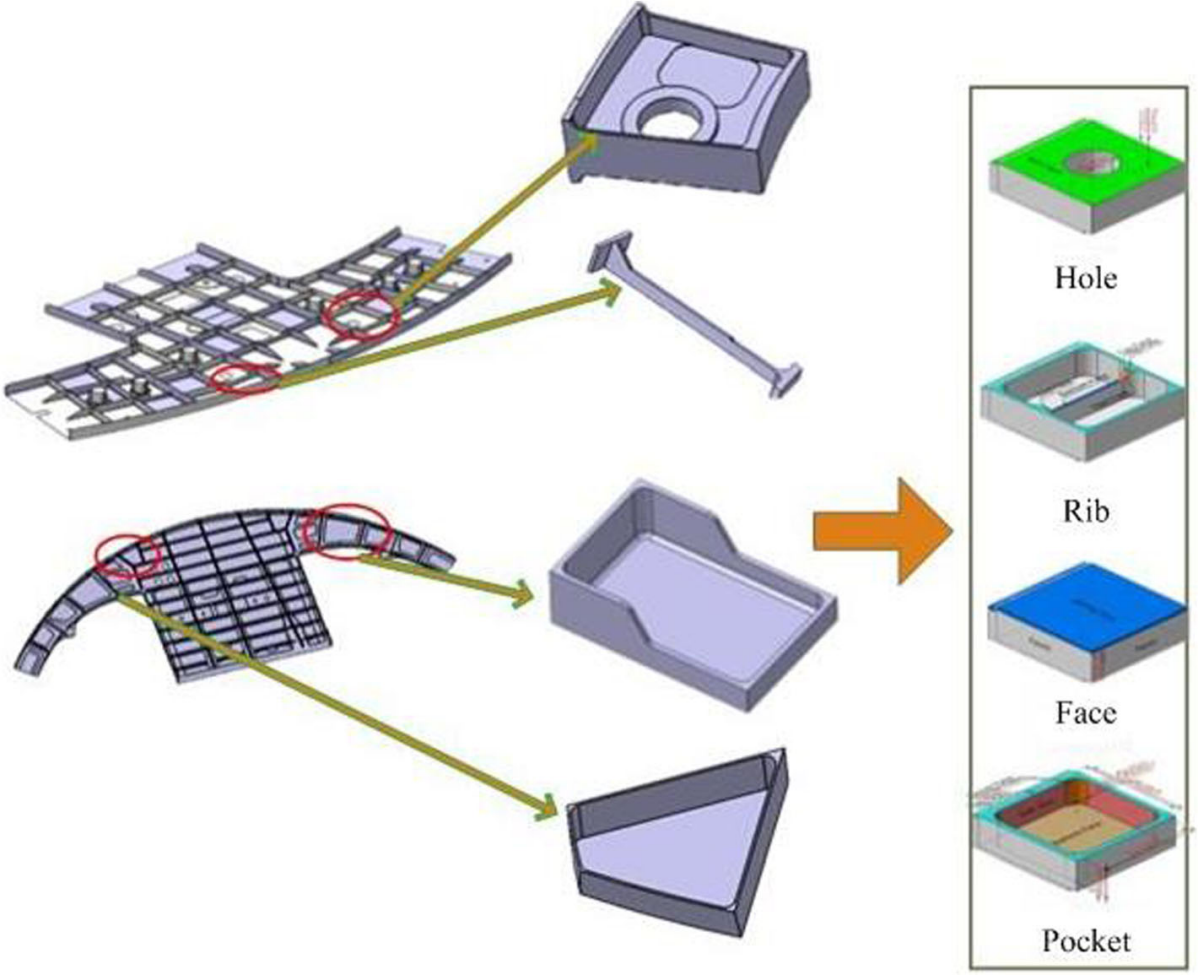
Typical machining features of thin-walled aircraft structural parts

As the carrier of process knowledge, machining features can obviously improve the efficiency and standardization of machining process preparation and reduce the influence caused by process experience of personnel, and thus improve the stability of processing quality. In feature-based process scheme, using machining features instead of the whole entity parts model can make information be expressed clearer and more structured. For complex thin-walled structures, it is basically composed of typical machining features such as pockets, ribs and holes, as shown in Fig. 3.

**Fig. 4:**
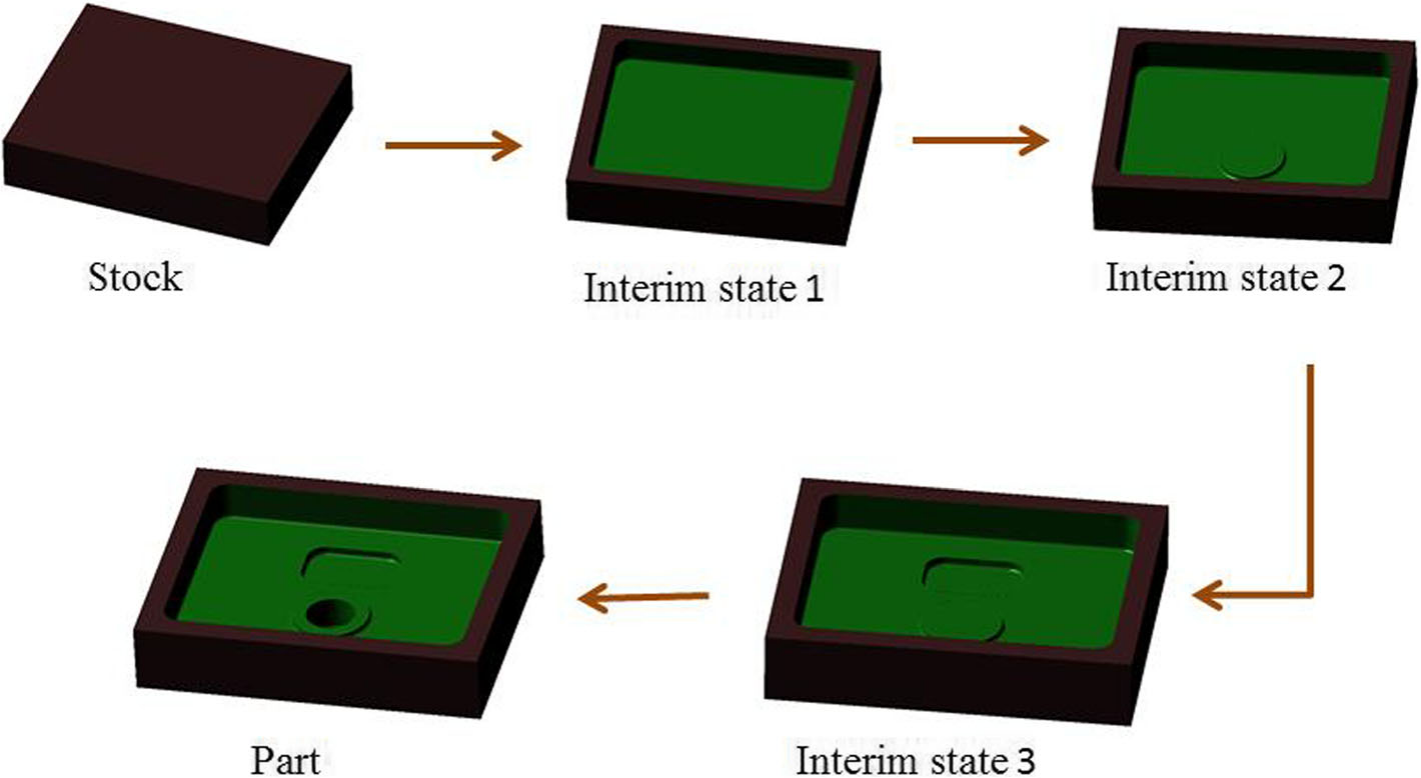
Diagram of machining feature interim state construction

Each machining feature corresponds to the specific machining operation in the forming process. Therefore, in order to process the stock into the final part, many machining operations are required. A corresponding interim state of machining feature will be formed after each machining operation is completed. The geometric shape of feature interim state will be changed according to the change of machining allowance. Each interim state of machining feature is the result of the last machining feature interim state, and the next machining interim state is produced after the corresponding machining operation. Figure 4 shows the construction process of the interim state of machining features in the machining process.

For the finishing process of thin-walled parts, the interim state geometry of machining features can be represented as the model geometry after rough machining minus the machining units of machined features:

1 $$ PG= RG-\sum \limits_{i=1}^k{FG}_i $$

In the formula *PG* denotes the interim state geometry of the current machining feature, *RG* denotes the model geometry after rough machining, *FG*_*i*_ denotes the geometry of the finishing machining unit of the *i-th* machining feature, *k* denotes the number of machined features.

#### Stiffness evaluation method

The surface stiffness of the machining feature is an important basis for the allocation of non-uniform allowance. How to evaluate the stiffness of each machining surface is a crucial issue. According to the relevant stiffness theory and practical experience, we can easily know that the thicker the side wall the better the stiffness and the larger the surface area the worse the stiffness. Based on the above two points, a surface stiffness index of machining feature *ε* is presented in this paper for the complex thin-walled structural parts, which can be shown as:

2 $$ \varepsilon =\frac{\delta }{A}\times {10}^4 $$

**Fig. 5:**
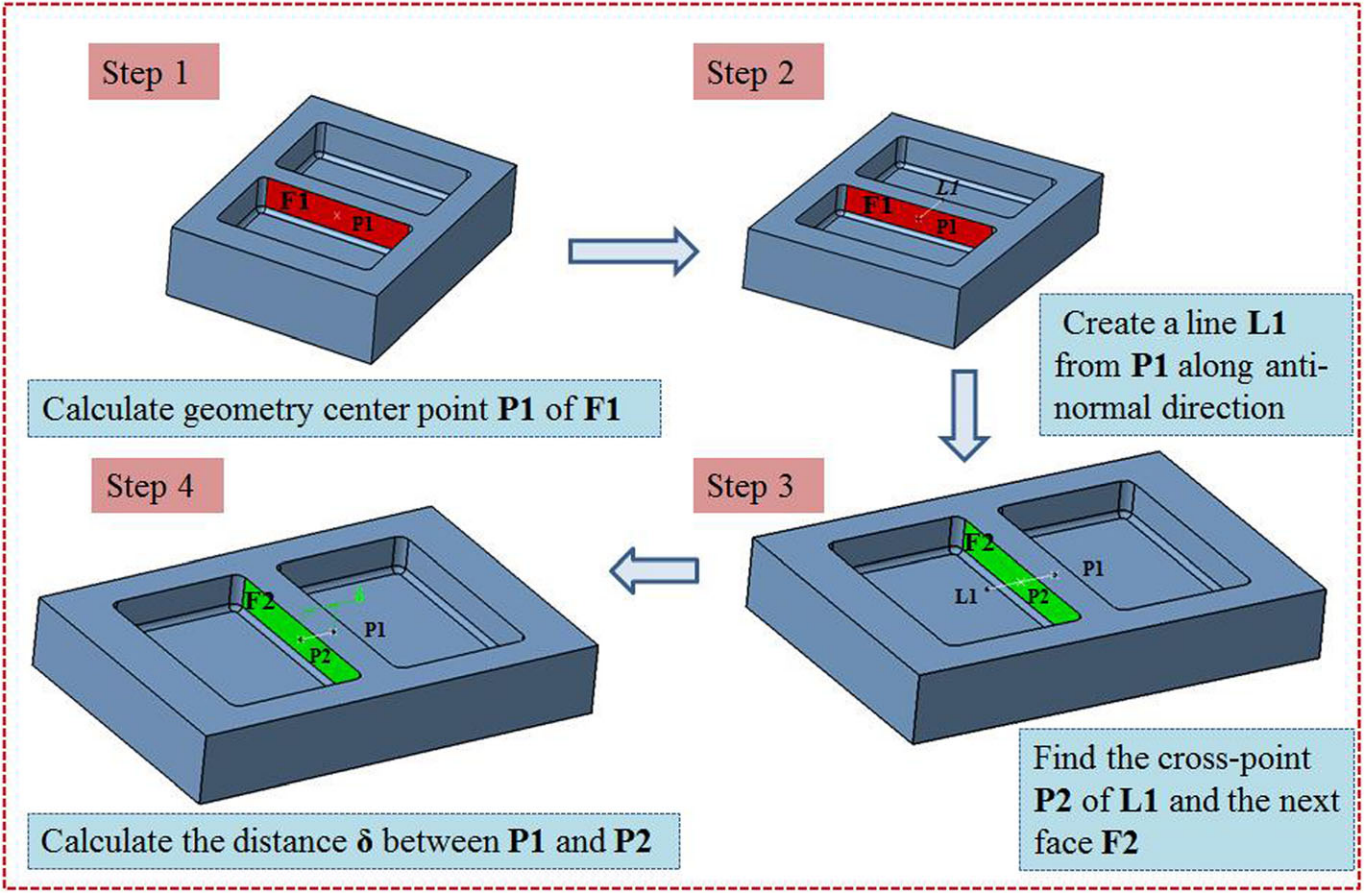
Wall thickness calculation process of machining surface

In the formula *δ* denotes the wall thickness of machining surface in the current interim state of machining features, *A* denotes the area of machining surface. The method for calculating the wall thickness of the machining surface follows the process shown as Fig. 5.

### Algorithm of non-uniform allowance allocation method

Based on the concept of surface stiffness index of machining feature, a non-uniform allowance allocation algorithm for finishing of thin-walled structural parts is proposed. The steps of the algorithm are as follows:

1. According to the geometric model of parts and the machining requirements, the reasonable range of machining allowance is set by the relevant technicians, that is the maximum machining allowance *Z*_*max*_ and the minimum machining allowance *Z*_*min*_;
2. Calculate the wall thickness *δ*_*i*_and area *A*_*i*_of each machining surface for the current machining feature. Then we can get the corresponding surface stiffness index *ε*_*i*_. Using all of the surface stiffness indexes, we can get the average stiffness index of the machining feature, which can be shown as:

3 $$ {\varepsilon}_{avg}=\frac{\sum \limits_{i=1}^n{\varepsilon}_i}{n} $$

In the formula *ε*_*avg*_ denotes the average stiffness index of the current machining feature, *n* denotes the number of machining surfaces in the machining feature.

(3) In order to equalize the stiffness of each machining surface, by comparing the stiffness index of each machining surface with the average stiffness index, the finishing allowance of each machining surface is adjusted appropriately, which can be represented by the following formula:

4 $$ {Z}_i=\left\{\begin{array}{c}{Z}_{min},{Z}_i<{Z}_{min}\\ {}{Z}_{oi}+\frac{\left({\varepsilon}_{avg}-{\varepsilon}_i\right){Z}_{oi}}{\varepsilon_{max}-{\varepsilon}_{min}},{Z}_{min}\le {Z}_i\le {Z}_{max}\\ {}{Z}_{max},{Z}_i>{Z}_{max}\end{array}\right. $$

In the formula *Z*_*i*_ denotes the adjusted allowance value, *Z*_*oi*_ denotes the original allowance value, *ε*_*max*_ denotes the maximum surface stiffness index of the current machining feature, *ε*_*min*_ denotes the minimum surface stiffness index of the current machining feature.

## Results and discussion

**Fig.6:**
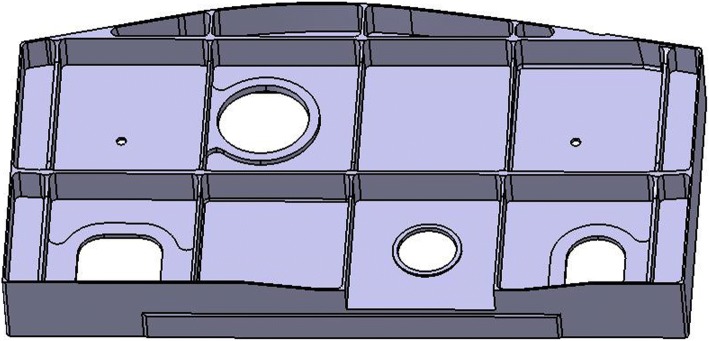
A typical thin-walled structural part

In order to validate the proposed non-uniform allowance allocation method, a thin-walled structural part shown as Fig. 6 is adopted as a case study.

### Implementation process

**Fig. 7:**
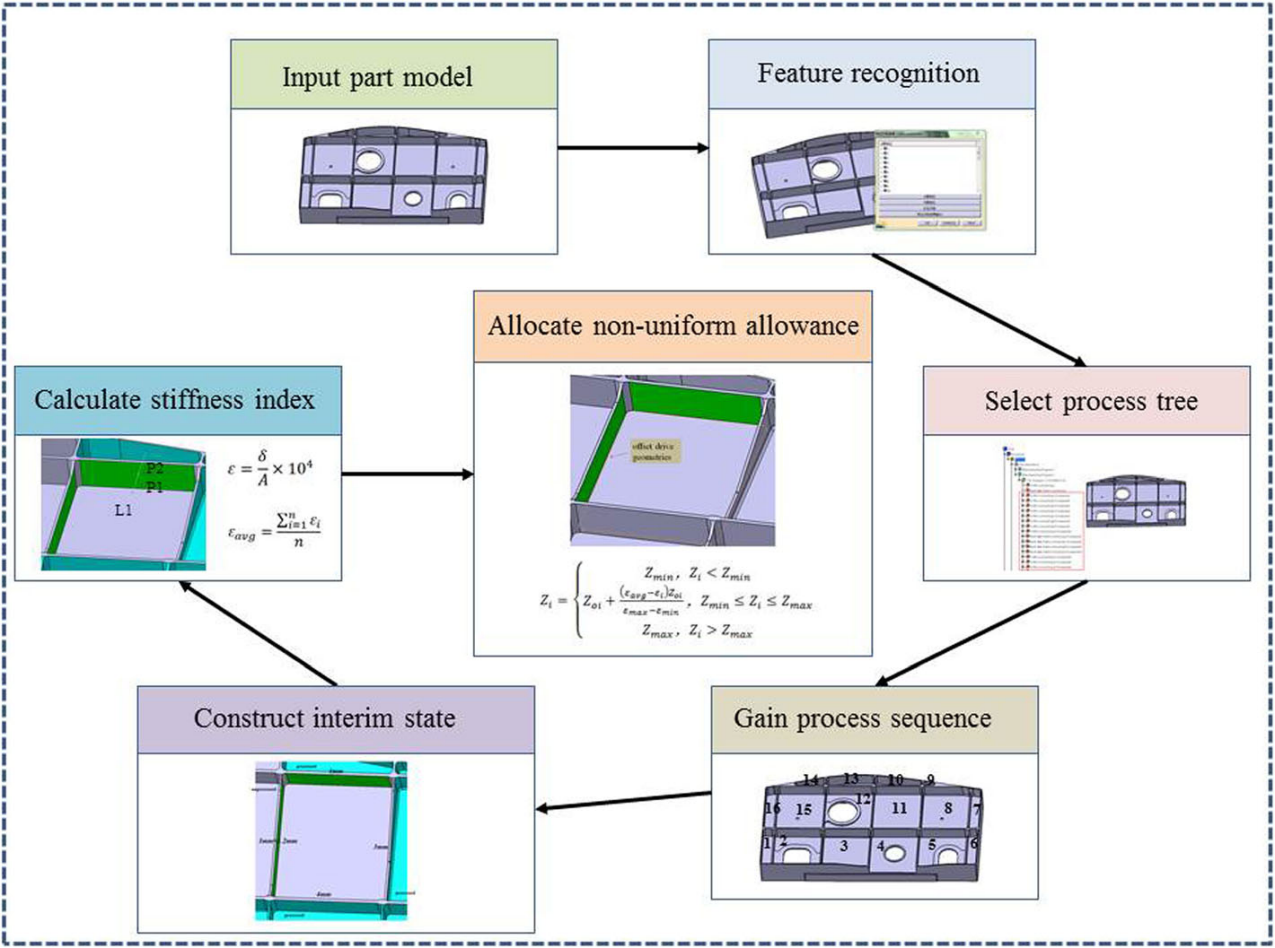
Process of non-uniform allowance allocation

The whole implemented process can be described as Fig. 7.

**Step 1:** Input the part model and execute the process of feature recognition. By analyzing the structural and machining characteristics of the part, divide the machining features into several classifications.

**Step 2:** Select process tree and obtain the machining operations related to the machining features. Then, we can find the corresponding relationship between machining operations and machining features according to the machining feature identifiers. And then according to the sequence of machining operations in the process tree, the machining sequence of machining features is obtained.

**Step 3:** According to the machining sequence of machining features, construct the interim machining states successively. The interim machining state geometry corresponding to the current machining feature is constructed by the current machining feature geometry overlying the finishing allowance. Then the interim machining state geometry corresponding to each machining feature can be obtained by analogy.

**Fig.8:**
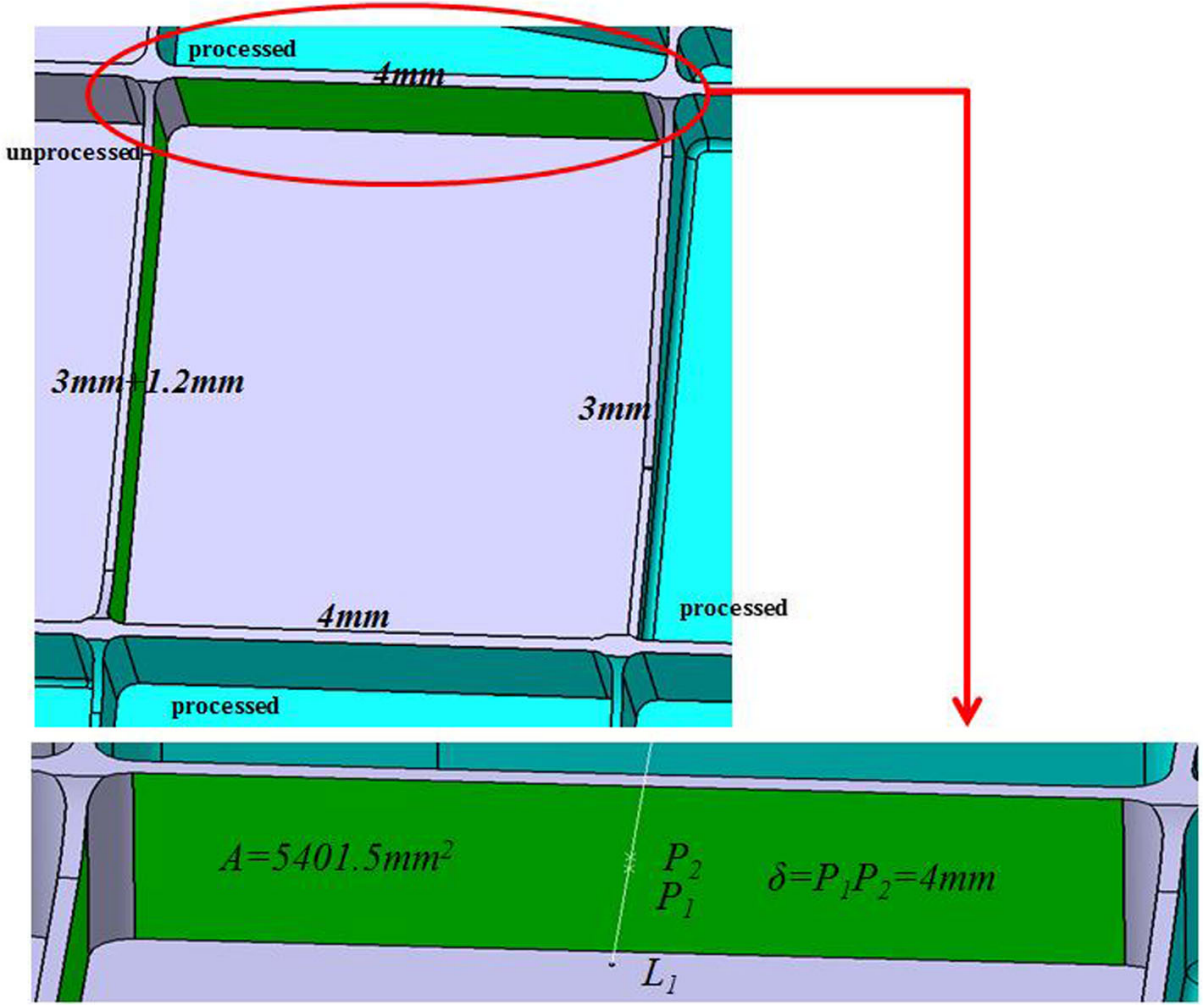
A pocket feature of the thin-walled part

**Step 4:** Aiming at the interim machining state geometry of each machining feature, calculate the stiffness index *ε*_*i*_ of each machining surface according to the method mentioned above. Take one pocket feature out of the thin-walled part to validate the allowance allocation process, which can be shown as Fig. 8.

Assuming that the allowance range of machining feature to be processed is 0.5 mm~ 2 mm and the initial uniform finishing allowance *Z*_*oi*_ = 1.2 mm according to the relevant machining process requirements. The side wall thickness corresponding to each machining surface can be calculated as follows: 4 mm, 3 mm + 1.2 mm, 4 mm, 3 mm. Then according to formulas (2), (3) and (4), we can calculate the corresponding stiffness index of each surface is 7.41, 8.53, 7.41, 9.95, the average value of stiffness index of machining feature is *ε*_*avg*_ = 8.33, and the allowance after adjustment of each machining surface is 1.63 mm, 1.11 mm, 1.63 mm, 0.43 mm.

**Step 5:** After obtaining the adjusted non-uniform allowance of each machining surface, the new machining drive geometries can be generated by offsetting allowance value based on the original ones.

### Simulation analysis

**Fig. 9:**
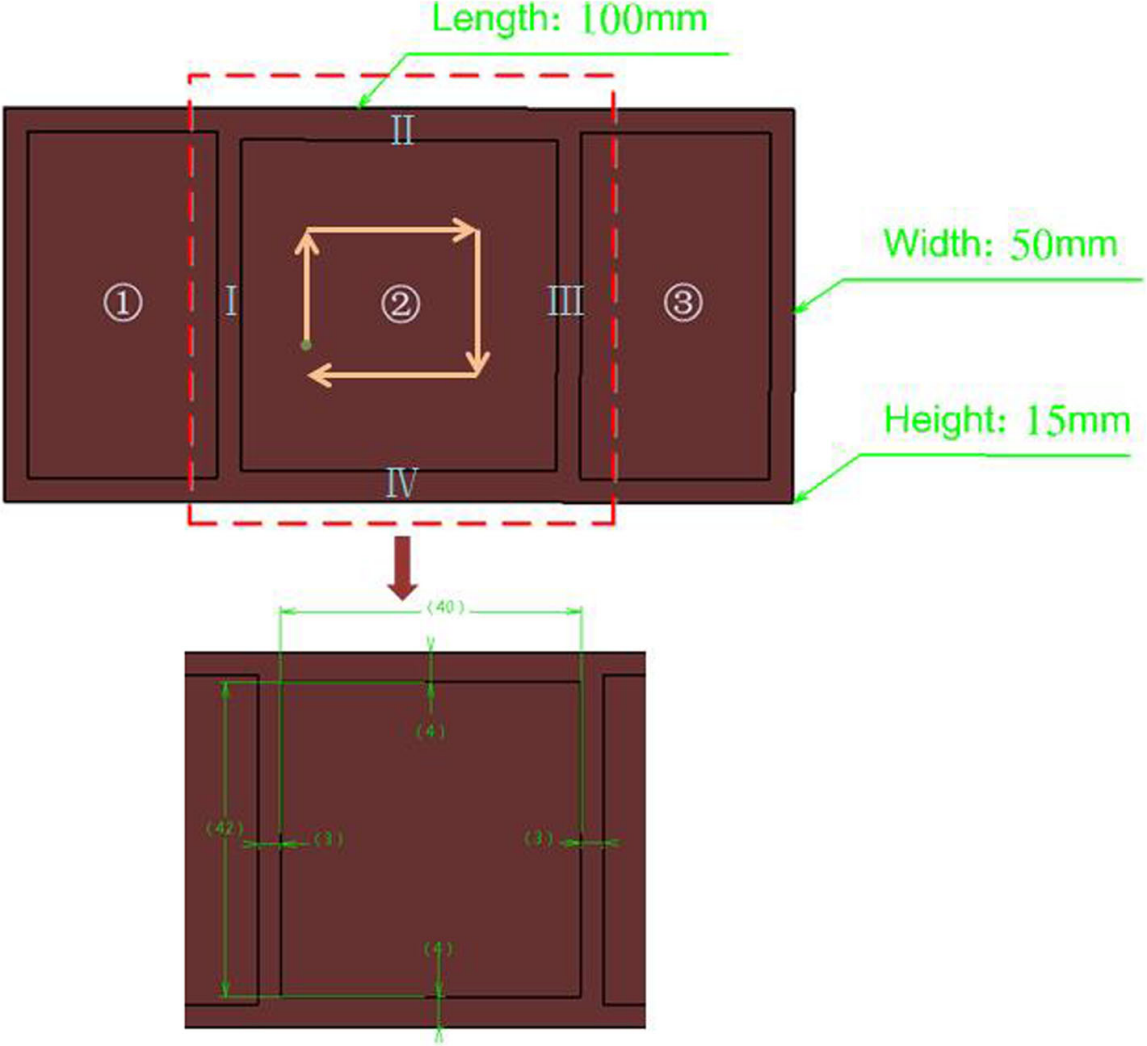
Example for simulation

To verify the effectiveness of the proposed method in improving the machining stiffness of parts, a thin-walled part with three pockets (shown as Fig. 9**)** is adopted to do the finite element simulation on ABAQUS. The basic information for the part is shown in the Table.1, and the machining sequence of the three pockets in the part is clockwise. Take the four walls of the middle pocket out to carry out finite element analysis. In the simulation, we set uniform distributed load at 500 Pa with three-terminal fixed constraint.

**Table. 1:** Basic information of the part

Material	Elastic modulus /GPa	Poisson’s ratio	Length /mm	Width /mm	Height /mm	Depth of pocket /mm
7075 Al	71.7	0.33	100	50	15	10

Assuming that the reasonable allowance range is 0.5 mm to 2 mm and the initial uniform allowance is 1.2 mm. According to the proposed method, the adjusted allowance values of the four walls are *Z*_*1*_ = 2 mm, *Z*_*2*_ = 0.87 mm, *Z*_*3*_ = 0.9 mm, *Z*_*4*_ = 0.9 mm.

**Fig. 10:**
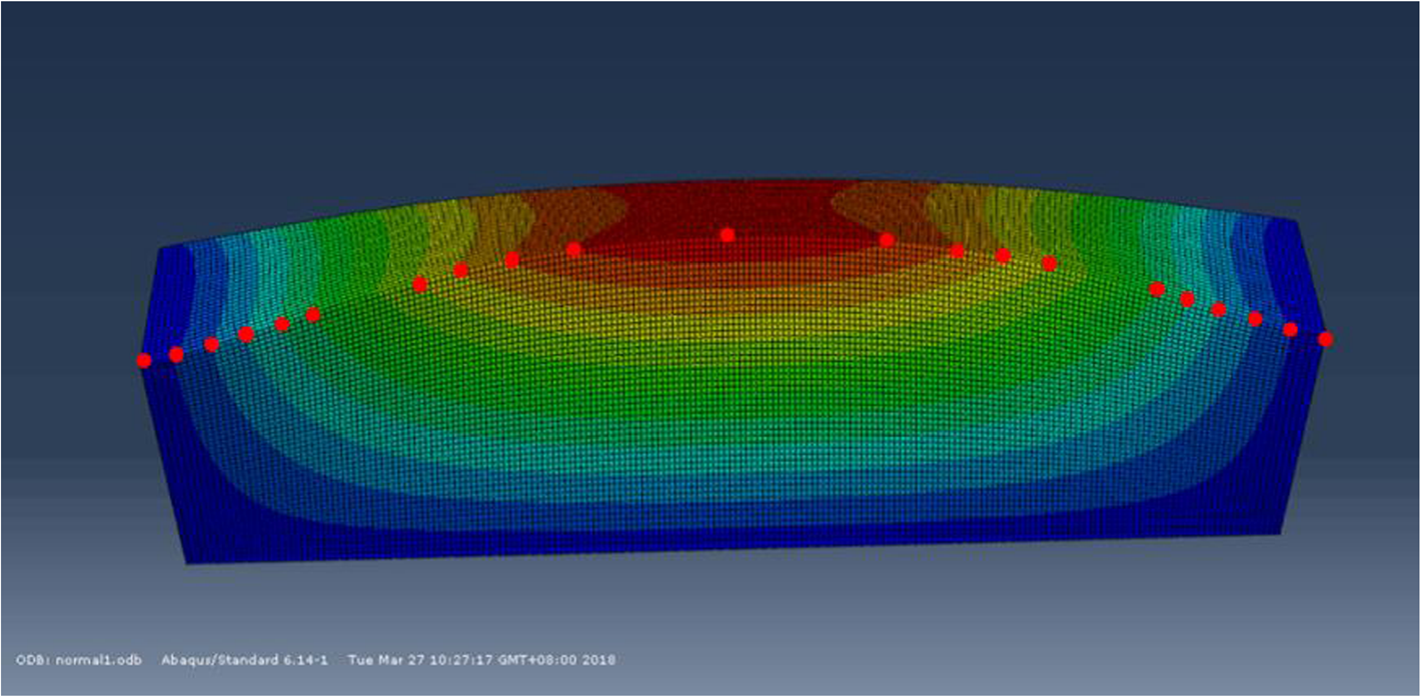
Simulation result of side wallI

**Fig. 11:**
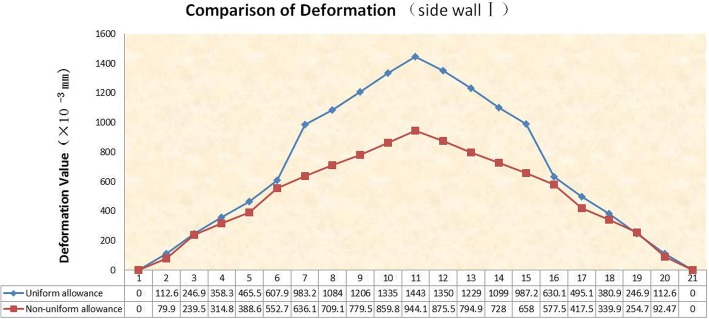
Deformation comparison of two methods

It is easy to know that the first side wall is the worst in stiffness by the proposed method. If the maximum value of its deformation is within the required range, the amount of deformation of the remaining three side walls is also within the required range. As shown in Fig. 10, select the 21 reference points along the horizontal direction to draw the deformation trend map of different positions of the first side wall. In the same way, we use the same load and constraint to simulate the deformation of the first side wall under the non-uniform allowance method. We can easily find that the maximum deformation is located at the middle point on the top surface of the side wall. It is obvious that the deformation at each reference point of the non-uniform allowance method is smaller than that of the uniform allowance method when compared the deformation of these two methods (shown as Fig. 11**)**, and the maximum deformation decreases from the original 1.443 mm to 0.944 mm, which means a reduction by 34.6%.

**Fig. 12:**
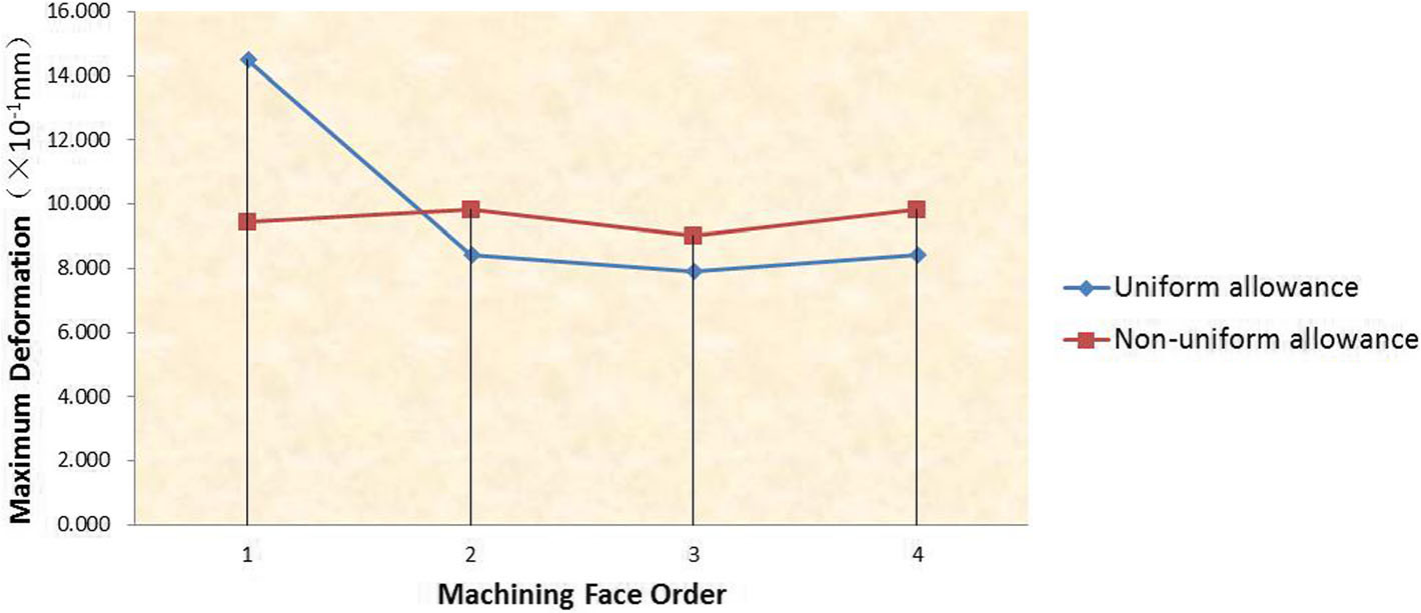
Maximum deformation of four walls

In order to fully compare the deformation of these two methods, the maximum deformation of the four walls of the pocket is given in Fig. 12. It can be seen from the diagram that the maximum deformation of the four walls is more uniform under the non-uniform allowance allocation method. Meanwhile, it is obviously that the maximum deformation of the four walls under the non-uniform allowance allocation method is much smaller than the maximum deformation of the first side wall under the uniform allowance allocation method.

## Conclusions

The machining of thin-walled parts is a crucial issue to NC machining. The traditional method will allocate uniform finishing allowance to each machining surface, which will easily leads to insufficient wall thickness and poor stiffness of some machining surfaces, resulting in excessive deformation of the final formed parts, or deduce machining efficiency for some machining features due to too thick remains. In order to solve this problem, a non-uniform allowance allocation method based on interim state stiffness of machining features for thin-walled parts finishing is proposed in this paper. According to the evaluation of interim state stiffness of each machining feature, the non-uniform allowance is allocated to ensure the stiffness requirement in the finishing process. The finite element simulation results reveals that the non-uniform allowance allocation method proposed in this paper can effectively guarantee the stiffness of the parts and reduce the deformation of the parts compared with the traditional uniform allowance allocation method.

Although the results are promising, there is still much room for improvements. In the above proposed method, the stiffness index is simplified to be proportional to the area and thickness of the machining surface. This may not be suitable for some complex compound features. Our future work will focus on exploring a more accurate stiffness evaluation method for complex machining features.
